# Tubulocystic Renal Cell Carcinoma: A Rare Renal Tumor

**DOI:** 10.15586/jkcvhl.2014.13

**Published:** 2014-09-01

**Authors:** Jasneet Singh Bhullar, Sandiya Bindroo, Neha Varshney, Vijay Mittal

**Affiliations:** 1 Department of Surgery, Providence Hospital & Medical Centers, Southfield, MI, USA; 2 Department of Pathology, University of Toledo, Toledo, OH, USA

## Abstract

Tubulocystic renal cell carcinoma of the kidney is a rare entity with less than one hundred cases reported so far. It was previously considered to have some similarities to various other renal cancers although this tumor has distinct macroscopic, microscopic and immuno-histochemical features. It is now a well-established entity in renal neoplastic pathology and has been recognized as a distinct entity in the 2012 Vancouver classification of renal tumors. This review aims to give an overview of tubulocystic renal cell carcinoma after extensive literature search using PubMed and CrossRef.

## Introduction

Kidney cancer is among the ten most common causes of cancer related deaths in adults (1). The National Institute of Health estimates around 63,920 new cases of kidney cancer and 13,860 deaths from this disease in 2014 (2). The common renal cell carcinomas of clear cell, papillary and chromophobe types account for 85–90% of the renal tubular malignancies and the remaining 10–15% includes a variety of uncommon sporadic and familial carcinomas, some of which have been recently described, along with a group of unclassified carcinomas. One of these tumors is extremely rare and is known as tubulocystic renal cell carcinoma.

Tubulocystic renal cell carcinoma of the kidney is a recently established entity in renal neoplastic pathology. It was first described by Pierre Masson in 1956 who described cystic neoplasm of the kidney with hobnail cells in the central region of the kidney (3). It was thought to be in collecting duct and was hence called carcinoma of Bellini (Collecting) duct. It was later found to be low grade and differ significantly in behavior when compared to classic type which was far more aggressive. Accordingly, it was termed as “low-grade collecting duct carcinoma”. In 1997 MacLennan et al. hypothesized that this tumor represented the low grade of the spectrum of collecting duct carcinoma (CDC), as it shares similar characteristics with the latter tumor (4). A recent study by Osunkoya et al. has shown that tubulocystic renal cell carcinoma is distinct from CDC at the molecular level (5). It received its current name in 2004 in a series of 31 cases presented in an abstract at the United States and Canadian Academy of Pathology meeting by Amin et al (6). Tubulocystic renal cell carcinoma was not included in the WHO 2004 classification. However, it was recognized as a distinct entity in 2010 by the American Joint Committee on Cancer. In 2012, it was included in the Vancouver classification of renal cancer (7).

## Search Criteria

We performed English literature search using Pubmed and Crossref on 10^th^ August 2014, which yielded more than 80 results of published material on Pubmed. The search terms used were “tubulocystic renal cell cancer” and “tubulocystic carcinoma pathology / metastasis / immunohistochemistry” and “uncommon cystic renal tumors”. We have critically analyzed and included most of the important case series, reports and previous reviews from 1970- August 2014 in our review. Cases appear to be focusing on tumor histology and differentiation with other similar subtypes of renal tumors. There has been a surge in the reports and reviews in the past 7 – 8 years indicating a recent interest among researchers in the study and management of this tumor. Here in, we review the literature about tubulocystic renal cell carcinoma.

## Clinical features

Clinically, tubulocystic renal cell carcinoma is a tumor of adults mostly presenting in the fifth and sixth decade with a wide age range, 29–94 years. It shows a strong male predominance with a male/ female ratio of 7:1. Reported tumors are more often left sided (8). Tubulocystic renal cell carcinoma is usually solitary; however, based on the literature, multifocality appears to be a common phenomenon in up to 23% of cases (^8-9^). They are less aggressive than other renal cell carcinomas. Patients are often asymptomatic, although they may present with abdominal pain, distension and hematuria. Most present with small tumors (pT1), however, occasional pT2 and pT3 lesions have been reported. They rarely progress, recur, or metastasize (10). In the vast majority of reports, this has been an incidental finding on autopsy, nephrectomy for a separate disease process, or imaging (6). Clinical characteristics reported in different studies have been explained in Table 1.

**Table 1:** Clinical characteristics of TCRC

Number of cases	Age range (years)	Sex	Size range (cm)	Location (kidney)	Nature of tumor (recurrence)	Metastasis	Ref
13	36-94	10(M) : 3(F)	0.5 – 8.5	R=6; L=7	Nil	1 case: local lymph nodes	9
20	36-87	16(M) : 4(F)	0.2 – 6.1	R>L	Only 1 case recurred	1 case: local 1 case: distant	17
11	30-80	11 (M)	1.7 – 7	Data not available	Nil	Nil	13
31	34-74	27(M): 4(F)	0.7-17	L>R	Nil	2 cases: local	6
6	29-83	3(M): 3(F)	1.9-4.0	R=5; L=1	Nil	1 case: local lymph node	18
5	29-70	4 (M)One is missing	5.1 – 6.7	R>L; 3	1 case: recurrence; 1 death	2 case: distant metastasis	19
1	33	M	5x2.7	L	Recurrence in peritoneum	Distant metastasis	12
1	43	M	-	R	-	-	15
1	28	F	12	R	Solitary	Local metastasis	20
4	30-74	3(M): 1(F)	1.9 – 14.5	L>R	No recurrence reported	No metastasis reported	21
3	50-70	3(M)	3.8-14.0		No recurrence reported	1 vascular invasion 2 perinephric adipose tissue	22
1	70	M	15.1x11.6x9.	L	No recurrence	Bone Metastasis	23
1	35	F	010x12	L	No recurrence reported	No metastasis reported	24
				**Summary**			
~98	29-94	M>F	0.2 – 17	L>R	Less chances of recurrence	Local and distant metastasis	

Only a few cases so far have been reported of metastases to lymph node, bone, pleura, and liver (11). Its relationship to collecting duct carcinoma is controversial, and recent studies have linked it with papillary RCC. Only one case of tubulocystic carcinoma with sarcomatoid features has been reported where the patient developed multiple peritoneal metastases and died 14 months after diagnosis (12).

## Gross Description

Grossly, it is usually solitary, although multifocal examples may be seen. The tumors show a variable size, ranging from 0.3 to 17 cm, with a mean of 4 cm. The tumors are well circumscribed and usually un-encapsulated involving mainly the renal cortex. The cut surface of tumor is white or gray often compared to “bubble wrap”. It reveals small multilocular cystic spaces reminiscent of the appearance of a ‘‘sponge’’ or ‘‘Swiss cheese’’ (9).

## Microscopic Description

**Figure 1.**
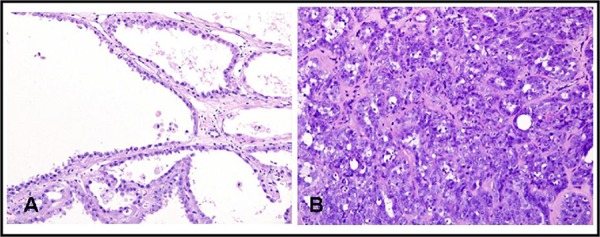
Microscopic appearance of tubulocystic renal cell carcinoma. A, hematoxylin and eosin (H & E) staining shows tubulopapillary pattern with cystic spaces lined with cells having hobnail appearance (x200); B, H & E shows tubular and tubulopapillary structures in a hyalinized and fibrous stroma (x200).

Microscopically, tubulocystic renal cell carcinoma is composed of tightly packed tubules and cysts measuring up to a few millimeters in diameter, separated by bland fibrous stroma. The lining cells are cuboidal to columnar and may have an attenuated appearance. Hobnail cells are commonly seen (Figure 1A & B). The cells have abundant eosinophilic or amphophilic cytoplasm and the nuclei are large and have prominent nucleoli. The nuclear grade generally corresponds to Fuhrman grade 3, but grade 2 or even grade 1 may be seen (8,13). Nuclear chromatin is evenly dispersed. A solid sheet pattern is absent (9). Occasional cells with low grade nuclear changes may be seen but they rarely predominate. Rarely minor areas with clear cell or papillary features are noted (^**17**^). Foam cells, calcospherites and hemosiderin encrustation are not found, unless there is an accompanying papillary renal cell carcinoma (4). Electron microscopy reveals abundant microvilli in most cells with a brush border appearance resembling proximal convoluted tubules. Admixed cells with short, sparse microvilli and complex cytoplasmic interdigitation, reminiscent of intercalated cells of collecting duct are also seen (8).

## Molecular characteristics

Immunohistochemistry demonstrates a poor relationship between tubulocystic renal cell carcinoma and collecting duct tumors. These tumors show expression of proteins of proximal convoluted tubules (CD10 and P504S), distal tubules (CK19) and intercalated collecting duct cells (parvalbumin). They also show vimentin, p53 and alpha methylacyl CoA racemase (AMACR) over-expression in contrast to CDC. High molecular weight cytokeratin (34BE12) is nearly always negative (5).

Gene expression microarray analysis by Yang et al. (9) demonstrated a unique molecular signature of tubulocystic renal cell carcinoma in comparison to other renal tumors and normal renal tissue. Clustering analysis of that data revealed tubulocystic renal cell carcinoma to be closely related to papillary RCC. Both types 1 and 2 dimensional clustering placed tubulocystic carcinoma between low and high grade papillary RCC. The most common karyotypic changes in papillary RCC are trisomy 7 and 17 with loss of Y chromosome. Yang et al (9) also reported that tubulocystic renal cell carcinoma showed gain of chromosome 17, but not chromosome 7. Thus, although tubulocystic renal cell carcinoma has been reported in association with multiple other renal cell tumor subtypes, it appears that there is a slight predominance for synchronous tubulocystic carcinoma and papillary tumors. These reports of coexistence thus raise an interesting question about presence of common predisposing factors for these histologically different tumors.

Diagnostic investigations play a very pertinent role as several differential diagnoses are to be considered. This is especially important since tubulocystic renal cell carcinoma may be associated with malignant behavior, that is, a high vigilance for potential metastatic disease. As the tumors have cystic elements, the radiological differential diagnostic spectrum is broad, including lesions that may be classified as a Bosniak type III or IV.

## Differential diagnoses

The clinical and pathological aspects of tubulocystic carcinoma are still not well studied. A combination of histology, electron microscopy and immunohistochemistry are required for proper diagnosis. The main differential diagnosis of tubulocystic carcinoma includes collecting duct carcinoma and other cystic tumors including cystic nephroma, multilocular cystic RCC, and oncocytoma (14). Cystic nephroma is characterized by larger cysts with hyalinized or fibrotic stroma. The cysts are lined by flat to attenuated cells with occasional hobnailing. However, unlike tubulocystic renal cell carcinoma, the nuclei are typically benign with absent or inconspicuous nucleoli. Cystic nephroma has a characteristic ovarian-like stroma between the cyst, which is often positive for estrogen and progesterone receptors. In addition, cystic nephroma occurs almost always in females, whereas tubulocystic renal cell carcinoma is more common in males. Multilocular cystic renal cell carcinoma is characterized by variably sized cystic spaces lined by flattened to cuboidal clear cells separated by fibrous stroma containing small groups of clear cells of low Fuhrman nuclear grade. Oncocytoma may have in a minority of cases prominent dilated tubules, which can resemble tubulocystic renal cell carcinoma, as they both have abundant eosinophilic cytoplasm and can have cells with prominent nucleoli. However, the nuclei in oncocytoma are round or, if irregular, have degenerative atypia; whereas in tubulocystic renal cell carcinoma, the nuclei are irregular without degenerative features. Collecting duct carcinoma and renal medullary carcinoma occur in the renal medulla; and demonstrate a poorly differentiated adenocarcinoma, inflammatory infiltration, frequent perirenal fat invasion, lymphovascular invasion, intraluminal mucin and high nuclear grade (5).

## Management and outcome

Radical nephrectomy is generally recommended, but partial nephrectomy may be performed for small tumors located in the superficial renal cortex. Sunitinib, a tyrosine kinase inhibitor, may exhibit a partial response or temporary effect for this tumor (15). Tubulocystic renal cell carcinoma of the kidney with sarcomatoid change has responded poorly to sorafenib (12). The antiangiogenic targeted therapeutic protocols such as VHL/HIF, RTK/MAPK and PI3K/Akt/mTOR seem to have no rationale of general recommendation (16). In the three largest series totaling 34 cases with follow-up, only 3 developed metastases to lymph node, bone and liver. In contrast, in a series of 3 cases by Al Hussain et al. (22), one was with vascular invasion and two were with invasion to perinephric adipose tissue. This study reported 2 cases with poorly differentiated areas consisting of collecting duct–like carcinoma areas, and one with focal high-grade features with marked nuclear atypia and prominent nucleoli. Apart from this, there is only one similar description in the literature. Bhullar et al. recently reported 1 case of tubulocystic renal cell carcinoma with tubulopapillary pattern and focal sarcomatoid areas that developed multiple peritoneal metastases, and the patient died 14 months after diagnosis (12). Limited work has been reported in this regard and it raises concern that tubulocystic renal cell carcinoma with poorly differentiated areas increases the risk of aggressive behavior above that of usual tubulocystic renal cell carcinoma.

## Conclusion

Tubulocystic renal cell carcinoma is a distinct group of renal carcinoma with specific macroscopic, microscopic and immunohistochemical findings. It appears to have a close relation to papillary carcinoma. Tubulocystic renal cell carcinoma should be considered in differential diagnosis of cystic kidney lesions, along with cystic nephroma, multilocular cystic renal cell carcinoma, oncocytoma with prominent tubules and cysts, and mixed epithelial and stromal tumor of the kidney. However, it is distinctly different from these entities with regard to radiological findings, including growth pattern, the presence of cystic components and also in their biological aggressiveness. Tubulocystic renal cell carcinoma with poorly differentiated areas increases the risk of aggressive behavior above that of usual tubulocystic renal cell carcinoma. To date, no established guidelines have been given for the management of this tumor. The examination of more cases is required to understand its biology and ascertain true prognosis and appropriate treatment.

## References

[1] Jemal A, Bray F, Center MM, Ferlay J, Ward E, Forman D. (2011). Global cancer statistics.. CA: A Cancer Journal for Clinicians.

[2] “Cancer of the kidney and Renal Pelvis – SEER Stat Fact sheets”..

[3] Masson P. (1970). Tumeurs Humaines 1955.. Human Tumors, Histology, Diagnosis and Technique.

[4] MacLennan GT, Farrow GM, Bostwick DG. (1997). Low-grade collecting duct carcinoma of the kidney: report of 13 cases of low-grade mucinous tubulocystic renal carcinoma of possible collecting duct origin.. Urology.

[5] Osunkoya AO, Young AN, Wang W, Netto GJ, Epstein JI. (2009). Comparison of gene expression profiles in tubulocystic carcinoma and collecting duct carcinoma of the kidney.. American Journal of Surgical Pathology.

[6] Amin MB, MacLennan GT, Gupta R (2009). Tubulocystic carcinoma of the kidney: clinicopathologic analysis of 31cases of a distinctive rare subtype of renal cell carcinoma.. Am J Surg Pathol.

[7] Srigley JR, Delahunt B, Eble JN (2013). ISUP Renal Tumor Panel. The International Society of Urological Pathology (ISUP) Vancouver Classification of Renal Neoplasia.. Am.J.Surg.Pathol.

[8] Amin MB, Maclennan GT, Gupta R (2009). Tubulocystic carcinoma of the kidney: clinicopathologic analysis of 31 cases of a distinctive rare subtype of renal cell carcinoma.. Am J Surg Pathol.

[9] Yang XJ, Zhou M, Ondrej H (2008). Tubulocystic carcinoma of the kidney. Clinicopathologic and molecular characterization.. Am J Surg Pathol.

[10] Azoulay S, Vieillefond A, Paraf F, Pasquier D, Cussenot O, Callard P (2007). Tubulocystic carcinoma of the kidney: a new entity among renal tumors.. Virchows Archiv.

[11] Bhullar JS, Varshney N, Bhullar AK, Mittal VK. (2013). A New Type of Renal Cancer—Tubulocystic Carcinoma of the Kidney: A Review of the Literature.. Int J Surg Pathol Int J Surg Pathol.

[12] Bhullar JS, Thamboo T, Esuvaranat- han K. (2011). Unique case of tubulocystic carcinoma of the kidney with sarcomatoid features: a new entity.. Urology.

[13] Azoulay S, Vieillefond A, Paraf F, Pasquier D, Cussenot O, Callard P (2007). Tubulocystic carcinoma of the kidney: a new entity among renal tumors.. Virchows Archiv.

[14] Srigley JR, Delahunt B. (2009). Uncommon and recently described renal carcinomas.. Mod Pathol.

[15] Mego M, Sycova-Mila Z, Rejlekova K (2008). Sunitinib in the treatment of tubulocystic carcinoma of the kidney.. A case report. Ann Oncol.

[16] Steiner P, Hora M, Stehlik J (2013). Tubulocystic renal cell carcinoma: Is there a rational reason for target therapy using angiogenic inhibition? Analysis of seven cases.. Virchows Arch.

[17] Zhou M, Yang XJ, Lopez JI (2009). Renal tubulocystic carcinoma is closely related to papillary renal cell carcinoma: implications for pathologic classification.. Am J Surg Pathol.

[18] Amin MB, Gupta R, Ondrej H (2009). Primary thyroid like follicular carcinoma of the kidney. Report of 6 cases of a histologically distinctive adult renal epithelial neoplasm.. Am J Surg Pathol.

[19] Hora M, Michal M, Hes O. (2008). Re: Rodolfo Montironi, Roberta Mazzuccelli, Antonio Lopez-Beltran, *et al*. Cystic nephroma and mixed epithelial and stromal tumour of the kidney: opposite ends of the spectrum of the same entity?. Eur Urol.

[20] Deshmukh M, Shet T, Bakshi G, Desai S. (2011). Tubulocystic carcinoma of kidney associated with papillary renal cell carcinoma.. Indian Journal of Pathology and Microbiology.

[21] Borislav AA, Drachenberg CB. (2013). Tubulocystic carcinoma of the kidney: a histologic, immunohistochemical, and ultrastructural study.. Virchows Arch.

[22] Al-Hussain TO, Cheng L, Zhang S, Epstein JI. (2013). Tubulocystic carcinoma of the kidney with poorly differentiated foci: a series of 3 cases with fluorescence in situ hybridization analysis.. Hum Pathol.

[23] Iakovleva G, Iakovlev V, Ordon M, Srigley J, Yousef GM. (2014). Tubulocystic Carcinoma of Kidney: a Challenging Diagnostic Entity Mimicking Multicystic Kidney and Presenting with Bone Metastasis.. Histopathology.

[24] Ishibashi Y, Koie T, Fujita N, Satoh T, Mikami J, Hatakeyama S, Ohyama C, Tobisawa Y, Yoneyama T. (2014). Tubulocystic renal cell carcinoma in the left kidney: a case report.. J Med Case Rep.

